# Targeting hypoxia signaling pathways in angiogenesis

**DOI:** 10.3389/fphys.2024.1408750

**Published:** 2024-04-25

**Authors:** Sara Monaci, Federica Coppola, Irene Filippi, Alessandro Falsini, Fabio Carraro, Antonella Naldini

**Affiliations:** ^1^ Cellular and Molecular Physiology Unit, Department of Molecular and Developmental Medicine, University of Siena, Siena, Italy; ^2^ Department of Medical Biotechnologies, University of Siena, Siena, Italy

**Keywords:** hypoxia, angiogenesis, endothelial cells, miRNAs, HIFs, exosomes, immune cells

## Abstract

Oxygen (O_2_) supply is constantly maintained by the vascular network for a proper tissue oxygenation. Hypoxia is the result of an increased O_2_ demand and/or decreased supply and is common in both physiological conditions and human diseases. Angiogenesis is one of the adaptive responses to hypoxia and is mainly regulated by the hypoxia-inducible factors, HIFs. These heterodimeric transcription factors are composed of one of three O_2_-dependent α subunits (HIF-1, HIF-2, and HIF-3) and a constitutively expressed O_2_-insensitive subunit (HIF-1β). Among them HIF-1α is the most characterized and its activity is tightly controlled. Under hypoxia, its intracellular accumulation triggers the transcription of several genes, involved in cell survival/proliferation, autophagy, apoptosis, cell metabolism, and angiogenesis. HIF pathway is also modulated by specific microRNAs (miRNAs), thus resulting in the variation of several cellular responses, including alteration of the angiogenic process. The pro-angiogenic activity of HIF-1α is not restricted to endothelial cells, as it also affects the behavior of other cell types, including tumor and inflammatory/immune cells. In this context, exosomes play a crucial role in cell-cell communication by transferring bio-active cargos such as mRNAs, miRNAs, and proteins (e.g., VEGFA mRNA, miR210, HIF-1α). This minireview will provide a synopsis of the multiple factors able to modulate hypoxia-induced angiogenesis especially in the tumor microenvironment context. Targeting hypoxia signaling pathways by up-to-date approaches may be relevant in the design of therapeutic strategies in those pathologies where angiogenesis is dysregulated.

## 1 Introduction

The maintenance and regulation of oxygen (O_2_) homeostasis plays a crucial role in shaping the destiny of cells. Hypoxia is the result of low or inadequate O_2_ levels in tissue, due to an increased O_2_ demand and/or decreased supply, and it is present in several physiological and pathological conditions (Corrado and Fontana, 2020). The systemic hypoxic response is a regulatory mechanism that orchestrates various cellular activities to maintain homeostasis when faced with low O_2_ levels. This response increases O_2_ delivery by acting at both transcriptional and post-transcriptional levels altering energy metabolism, increasing cell motility, therefore promoting cellular adaptation, and enhancing the number of red blood cells or blood vessels. The key mediator of the adaptive response to hypoxia is the hypoxia‐inducible factor (HIF) family of transcription regulators. Indeed, HIF triggers the transcription of a multitude of genes associated with various biological processes, including glucose metabolism, cell survival, proliferation, apoptosis, and angiogenesis (Pugh and Ratcliffe, 2017; Nakayama and Kataoka, 2019; Lee et al., 2020). Notably, hypoxia promotes the development of blood vessels by upregulating various pro-angiogenic pathways that play crucial roles in the biology of endothelial cells, stromal cells, and vascular support cells (Liu et al., 2023).

The regulation of angiogenesis through hypoxia represents a vital element within the homeostatic regulatory mechanisms, connecting cardio-pulmonary-vascular O_2_ supply to the metabolic demand within local tissues (Carmeliet, 2003). Angiogenesis is a complex process that consists of several distinct steps including: a) degradation of the basement membrane by the production of proteases; activation and migration of the endothelium; b) proliferation of endothelial cells (ECs); c) formation of tube-like structures and capillary tubes (Li et al., 2018). However, hypoxic regulation of blood vessels is not only limited to homeostatic processes, but it has been associated with tumor angiogenesis. In tumors, the competition among actively proliferating cells restricts the availability of O_2_ and nutrients, while the diffusion of metabolites is inhibited by elevated interstitial pressure (Gupta et al., 2017). In response to intratumoral hypoxia, tumor cells generate angiogenesis-stimulating factors that trigger the development of a new blood supply from the existing vasculature. This process is critical for the survival and proliferation of tumor cells in a hostile microenvironment (Semenza, 2000; Lv et al., 2017). However, in tumors, neovessels are often abnormal, immature, and leaky and they are either insufficient or excessive depending on the tumor type (Carmeliet, 2005). This system provides tumor blood flow that expands rapidly, providing nutrients and O_2_ for thriving cancer cells (Schiliro and Firestein, 2021). However, an increase in the number of cells results in a higher demand for O_2_ and growth factors, exacerbating hypoxia that stimulates angiogenesis to alleviate the hypoxic condition. Consequently, the neoplastic tissue develops an excess of vasculature, which, even if dysfunctional, is providing enough O_2_ and nutrients to sustain tumor progression (Jiang et al., 2020). Pathophysiological angiogenesis is a complex and finely tuned process that is regulated by hypoxia, which could affect not only endothelial cells, but also other components of the tissue microenvironment. These include a multitude of other cell types, soluble factors, extracellular matrix-related molecules, which are contributing to the angiogenic process. In this review pathologic aspects of hypoxia-related angiogenesis will be treated in the context of tumor angiogenesis, although other pathologies characterized by aberrant angiogenesis are also known (Bosisio et al., 2018; Rezzola et al., 2020; Amato et al., 2022). Overall, targeting hypoxia signaling pathways by up-to-date approaches may be important in the development of therapeutic strategies in those pathologies where angiogenesis is uncontrolled.

## 2 The hypoxia-inducible factors

**FIGURE 1:**
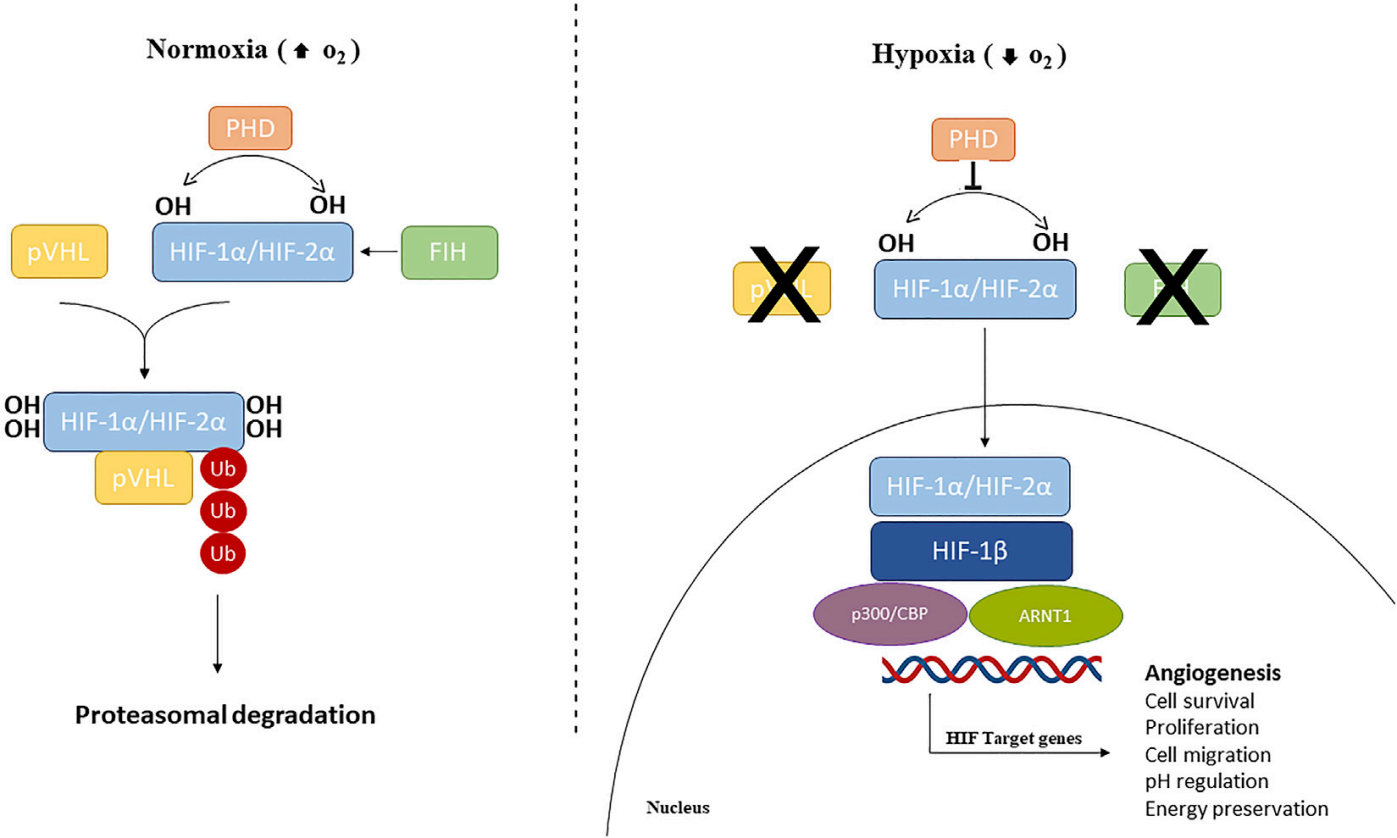
Hypoxia-inducible factors (HIFs) signaling pathway. Under normoxic conditions, HIF1/2 are hydroxylated by prolyl hydroxylase domain (PHD)-containing enzymes. Hydroxylated HIFs are degraded in the proteasomes by von Hippel-Lindau tumor suppressor protein (VHL) via polyubiquitination. During hypoxic conditions, PHDs and FIH are inhibited and HIF-α subunits are translocated into the nucleus, where can dimerize with HIF1-β, recruit p300 and CBP, and ultimately, bind to HREs at target genes to cause activation.

Oxygen levels are sensed by the human body by the master regulator of the cellular response to hypoxia, HIF, along with the 2-oxoglutarate (2-OG)-dependent oxygenase prolyl-hydroxylases (PHDs), the NAD(P)H oxidase family of enzymes that reduce reactive O_2_ species (ROS), O_2_ sensitive ion channels, and the electron transport chain (Arsham et al., 2003; Giaccia et al., 2004). HIF is a family of transcription factors consisting of a heterodimer of a constitutively expressed subunit, HIF-β, and an O_2_-regulated subunit, HIF-α (Semenza, 2007; Chao et al., 2021). Under normoxic conditions, HIF-α subunits (HIF1-α, HIF2-α, or HIF3-α) have a very short half-life since cells continuously synthesize and degrade HIF-α protein due to hydroxylation by the PHDs at specific proline residues (Pro564 on HIF1-α, Pro530 on HIF2-α, and Pro490 on HIF3-α) in the O_2_-dependent degradation domain. This hydroxylation event triggers a ubiquitination reaction facilitated by the E3 ubiquitin ligase Von Hippel–Lindau protein (pVHL), leading to HIF-1α proteasome-mediated degradation (Jaakkola et al., 2001; Maynard et al., 2003; Nakazawa et al., 2016). HIF-α subunits can also be hydroxylated by the FIHs (factor-inhibiting HIF-1α) proteins, a group of asparagine hydroxylase enzymes (Asn803 on HIF1-α and Asn851 on HIF2-α), that are dependent on the O_2_ concentration. Under normoxic conditions, FIHs prevent HIF-1α binding with its co-activators (p300/CBP) and therefore inhibit HIF transcriptional activity (Lando et al., 2002; Albanese et al., 2020). When the O_2_ availability is impaired, HIF-α hydroxylation is inhibited and HIF-α is stabilized. Therefore, the HIF-α subunit translocates to the nucleus where it interacts and dimerizes with the HIF-β subunit and the coactivators (p300/CBP). This heterodimeric complex induces the induction of target genes containing hypoxia-responsive elements (HREs) in their promoters, resulting in their transcriptional regulation (Javan and Shahbazi, 2017) (Figure 1). More than 100 genes have been identified as targets of HIF-1, including those encoding pro-angiogenic cytokines such as vascular endothelium growth factor (VEGF), platelet-derived growth factor (PDGF), and angiopoietin 1 (Ang-1) (Ke and Costa, 2006; Samanta et al., 2017), and thus HIF-1 is considered a master regulator of angiogenesis.

Of note, several O_2_ independent factors and signals, along with hypoxia, are able to modulate HIF-1α protein stability and thus to affect angiogenesis, including calcineurin A and calcium signaling, PI3Ks (Phosphoinositide 3 Kinases), FOXO4 transcriptional programs, RACK1 and several miRNAs (which are discussed below) (Koh et al., 2008).

## 3 HIFs role in endothelial cells in health and disease

Oxygen availability is essential to coordinate blood vessel growth with the metabolic demands of growing tissues, in both physiological and pathological contexts. Indeed, one of the best characterized and well-studied responses to hypoxia is the stimulation and induction of angiogenic factors, which lead to the formation and growth of new blood vessels (Chen et al., 2009). Thus, hypoxia-related angiogenesis has been considered as a major contributor to solid tumor growth, infiltration, and metastasis. Thus, the study of angiogenesis in this context has risen attention, and targeting tumor angiogenesis has emerged as a key method for solid tumor treatment (Cella et al., 2022). Of interest, angiogenesis, either in tumor, inflammatory and physiological setting, strictly involves HIFs, and in particular HIF-1. Indeed, HIF-1α, but not HIF-2α, has been demonstrated to be essential for endothelial cell (EC) cord formation as well as for the hypoxic-dependent pathway mediating EC survival. This suggests that HIF-1α may serve as a therapeutic target of the tumor microenvironment not only in tumor cells but also in stromal-infiltrating cells, including EC (Calvani et al., 2006). More recently, it has been demonstrated that ECs switch from HIF-1 to HIF-2 to adapt to prolonged hypoxia and that HIF-1α and HIF-2α mRNA stability differences contribute to the HIF-1/HIF-2 transitional switch. HIF-1 governs the acute adaptation to hypoxia, whereas HIF-2 activity begins later, creating the above-mentioned switch between these two HIF proteins (Bartoszewski et al., 2019). Of interest, the overexpression of HIF-1α has been associated with an induction of autophagy, during prolonged hypoxia, responsible for a reduced EC viability (Wu and Chen, 2015). However, ECs are heterogeneous and differentially reactive to hypoxia. EC in bone marrow from multiple myeloma patients shows a hypoxic phenotype, even under normoxic conditions, suggesting that ECs from tumor vessels are different from those of quiescent healthy vessels (Muz et al., 2015; Albanese et al., 2020). Moreover, ECs from multiple myeloma patients are more resistant than healthy EC in terms of cell viability, when exposed to prolonged hypoxia. ECs from tumor vessels also show a different metabolic response to prolonged hypoxia in terms of glucose consumption and transport as tumor-associated ECs have a higher glycolytic rate than normal EC and rely on glycolysis, as the main source of ATP production (Filippi et al., 2018; Rohlenova et al., 2018). Tumor-associated ECs increase glycolysis by upregulating VEGF with cyclooxygenase 2 (COX2) together with lactate accumulation under hypoxic conditions. Lactate acts as a signaling messenger able to stimulate angiogenesis via the VEGF pathway in a HIF-1α and PI3K/AKT-dependent manner (Ruan and Kazlauskas, 2013; Zhang et al., 2018).

## 4 Involvement of immune cells in the regulation of hypoxia-related angiogenesis

The pro-angiogenic activity of HIF-1α is not restricted to ECs, as it also affects the behavior of other cell types, including inflammatory and immune cells. T lymphocytes exert an angiogenic influence and secrete VEGF with important implications in the angiogenesis-inflammation crosstalk (Freeman et al., 1995; Mor et al., 2004). Hypoxic T cells may affect angiogenesis directly through the release of VEGF, but they also influence angiogenesis through adaptor proteins, such as p66Shc, which mediate VEGF expression under hypoxia (Naldini et al., 2010). A growing body of evidence also highlights the dynamic role of regulatory T lymphocytes (Tregs) in angiogenesis. Tregs can be pro- or antiangiogenic, depending on tissues and diseases: they promote angiogenesis directly by increasing the expression of VEGF and/or IL-10 or indirectly, through their effect on other immune cells. However, Tregs inhibit angiogenesis by inducing EC apoptosis via a cell contact-mediated process through DLL4 Notch and TNFR1 signaling or indirectly by modulating CD34^+^-circulating angiogenic cells via CCL5/CCR5 pathway (Lužnik et al., 2020). In the context of hypoxic tumor tissue, tumor-associated macrophages (TAMs) have been associated with increased angiogenesis and decreased survival in clinical specimens (Moeller et al., 2004; Cheng et al., 2021). Their role in angiogenesis has been particularly related to their ability to sense hypoxia in avascular areas of tumors and secrete pro-angiogenic factors, including VEGF and TNF-α (Kzhyshkowska et al., 2014). In the tumor microenvironment, these and other factors shape the TAM phenotype and skew them toward tumor-supportive M2-polarized macrophages, although M1-polarized TAM with anti-tumor activity was also reported in several types of cancer (Forssell et al., 2007). Neutrophils can also directly contribute to angiogenesis due to the release of cytokines and chemokines such as TNFα, IL-1β and VEGF, especially in the endometrium (Heryanto et al., 2004; Guo et al., 2021). In the context of tumor angiogenesis, neutrophils infiltrate the tumor tissue and contribute to tumor progression as reported for adenocarcinoma and melanoma (Tazzyman et al., 2009). Finally, several studies have shown the involvement of dendritic cells (DCs) in the angiogenic process due to their ability to express a wide array of pro- and anti-angiogenic mediators. That might have a significant role in those physiopathological settings characterized by DC activation and angiogenesis, including inflammation, wound healing, atherosclerosis, and tumor growth (Ormandy et al., 2006; Perrot et al., 2007; Bosisio et al., 2018). DCs release canonical angiogenic growth factors that act directly on the endothelium by interacting with specific receptors on the EC surface. In the mouse, DCs mediate the VEGF-dependent vascular growth in reactive lymph nodes (Webster et al., 2006). Still in the mouse, DCs show an hypoxic phenotype either in the spleen and in the bone marrow (Barroeta Seijas et al., 2022). Moreover, DCs are a significant source of chemokines, especially those able to modulate angiogenesis by direct or indirect mechanisms of action. In particular, DCs express the pro-angiogenic chemokines CXCL8 and CCL2 that induce angiogenesis by a direct action on ECs, whereas the CXC family of chemokines inhibit angiogenesis (Sozzani, 2005; Strieter et al., 2006). More interestingly, hypoxia enhances VEGF expression in either immature and mature DCs and hypoxic DCs express higher level of CXCR-4 receptor, with important implications in the tumor microenvironment and inflammation (Schioppa et al., 2003; Monaci et al., 2020; Monaci et al., 2021). Indeed, VEGF plays a role in reducing the number of mature DCs promoting the expansion and accumulation of immature tolerant DCs, and eventually causing the polarization of DCs towards Th2 or T regulatory (Treg) induction. These newly acquired characteristics of DC, contribute to the evasion of tumors from the immune response and have led to the identification of a new subtype of DCs known as Tumor-associated regulatory dendritic cells (regDCs) (Ma et al., 2012; Shurin et al., 2013). Together this highlights that immune cells contribute to the regulation of angiogenesis, either in a hypoxia-dependent or independent manner.

## 5 Hypoxia-associated miRNAs and angiogenesis

Recent studies have highlighted the role of microRNAs (miRNAs) during hypoxia providing a new and interesting link between hypoxia and the regulation of angiogenesis (Gee et al., 2010; Pocock, 2011). miRNAs are small nonprotein coding RNA molecules responsible for regulating mRNA stability and translation. They bind to the 3′UTR (3′ untranslated region) of mRNA, leading to a decrease in protein levels (Hastings and Krainer, 2001). Given that miRNAs decrease the protein output from existing transcripts, they are ideal candidates for controlling HIF expression during hypoxia. Among them, less than ten miRNAs have been demonstrated to affect HIF expression, with only three targeting directly HIF mRNA (Madanecki et al., 2013; Greco et al., 2014). Of note, HIF is responsible for the transcription of the so-called angiogenic “hypoxiamiRs” such as miR-210 (Huang et al., 2009), which is consistently and significantly induced by hypoxia. miR-210 targets the receptor tyrosine kinase ligand ephrin-A3 (EFNA3) which plays a critical role in the differentiation and migration of HUVEC in response to VEGF, by promoting angiogenesis (Maynard et al., 2005; Camps et al., 2008; Liu et al., 2012). More recently, miR-210, together with miR-424, has been found to be involved in HIF-1α on the angiogenesis under hypoxia not only in HUVEC but also in human dermal microvascular EC (HDMECs). In this context, both miR-210 and miR-424 are induced by hypoxia and target a splice variant of VEGFR1 (sFLT1), which functions also as a receptor on the surface of cells that VEGF binds to. Mechanistically, HIF-1α binds the promoter region of miR-210 and miR-424 to activate their transcription, while miR-210/miR-424 bind sFLT1 3′-UTR to suppress its expression suggesting that HIF-1α/miR-210/miR-424/sFLT1 axis modulates the angiogenesis in HUVECs and HDMECs upon hypoxic condition via VEGF signaling (Zhao et al., 2022). Another miRNA, miR-433, directly targets HIF1-α. However, miR-433 is downregulated in hypoxia-exposed HUVEC suggesting that the hypoxic reduction of this miRNA could promote HIF-1 signaling (Zhang et al., 2017). Most HIF-1-dependent miRNAs are also HIF-2-dependent in HUVECs under hypoxia. According to the next-generation validation system, there are six miRNAs dependent on both HIF isoforms, including miR-210-3p, miR-520d-3p, miR-4745-5p, miR-98-3p, miR-139-5p, and miR-6789- 5p. However, HIF-2 specifically governs the expression of several miRNAs providing an important level of miRNA-driven control in the hypoxia adaptive pathways in EC (Moszyńska et al., 2022).

## 6 Role of exosomes in angiogenesis associated with hypoxia

In a hypoxic microenvironment where ECs crosstalk with other cell types, exosomes play a crucial role by transferring bioactive cargos. Exosomes are small, single membrane extracellular vesicles derived from cells exhibiting various sizes and origins, not capable of replicating and without a functional nucleus (Di Bella, 2022). Exosomes have been considered to be an important mediator of cell-cell communication, proliferation, and differentiation by transferring various bio-active cargoes such as mRNAs, microRNA, proteins, and lipids, from one cell to another (Sun et al., 2020). Based on these and other properties, exosomes are being developed as therapeutic agents in multiple disease models (Ortega et al., 2022). While the application of exosomes shows promising effects in promoting angiogenesis in various animal models (Ma et al., 2018; Zhang and Li, 2020), unmodified exosomes alone demonstrate only moderate therapeutic efficiency and need to be enhanced by either genetic modification or engineering tools. However, exosomes with overexpression of HIF-1α in mesenchymal stem cells (MSCs), induced angiogenesis in the Matrigel plug assay via the expression of the Notch ligand Jagged1, with potential applications for the treatment of ischemia-related disease (Gonzalez-King et al., 2017). More recently, Sun et al. demonstrated that HIF-1α-overexpressed MSCs-derived exosomes in ischemic heart, rescue the impaired migratory ability, angiogenic function, and proliferation of hypoxia-injured HUVECs. In addition, exosomes overexpressing HIF-1α show a robust cardioprotective effect on myocardial infarction heart by promoting neovessels formation in the ischemic border zone (Sun et al., 2020). The potential therapeutical properties of hypoxic MSCs-derived exosomes in inducing angiogenesis were demonstrated also in other models including fracture healing, endometriosis, and spinal cord injury (Umezu et al., 2014; Qiu et al., 2019; Zhang et al., 2019). Of interest, MSCs are involved in the formation and modulation of tumor stroma and in interacting with tumor cells, partly through exosomes. Hypoxic condition within the tumor environment enhances the release of tumor-derived exosomes (TEX) and act as a potent trigger for the communication between cancer and ECs. After the uptake by ECs, TEX transfers molecular information, which promotes their adhesion, proliferation, migration, tube formation, and as a result pathological angiogenesis (Gluszko et al., 2019). In breast cancer, MSC-derived exosomes induce a significant and dose-dependent decrease in the expression and secretion of VEGF through the modulation of the mTOR/HIF-1α signaling axis, suggesting an important potential therapeutic target for this type of tumor (Pakravan et al., 2017). Over the past few years, carbonic anhydrases (CAs) have also been found to be involved in the regulation of angiogenesis via exosome release. CAs belong to zinc metalloenzymes that catalyze the reversible hydration of carbon dioxide into bicarbonate and protons, and they play a critical role in maintaining the cell pH homeostasis (Pastorek et al., 1994). Among human CAs isoforms, CA-IX is a transmembrane protein localized on the surface of normal cells including the stomach, duodenum, small intestine, and gallbladder. On the contrary, CA-IX overexpression is associated with a variety of solid cancers including melanomas (Chafe et al., 2019; Peppicelli et al., 2020). CA-IX is induced by HIF-1α and has been found to be highly expressed in invasive melanomas with only hypoxia-induced exosomes expressing CA-IX (Venturella et al., 2023). In addition, CA-IX-expressing exosomes released from hypoxic renal cell carcinoma cells promote angiogenesis and migration of HUVEC, suggesting the involvement of this enzyme in cancer progression (Horie et al., 2017).

## 7 Discussion and conclusion

**FIGURE 2:**
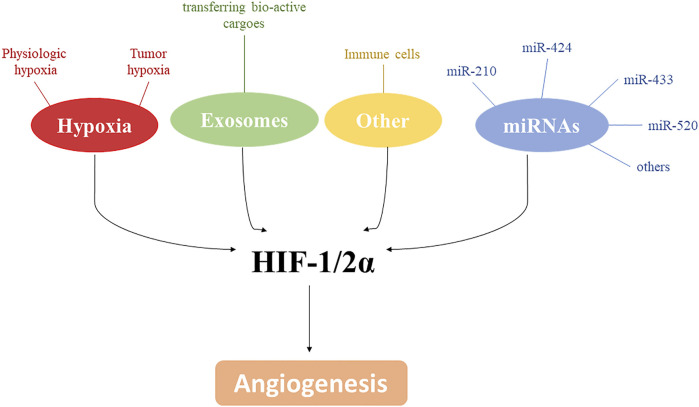
HIF-1/2α and angiogenesis. Along with hypoxia, several mediators, including exosomes, miRNAs and immune cells, are involved in angiogenesis modulation by affecting directly or indirectly HIF-1/2α.

The control of angiogenesis by hypoxia is a crucial component of the homeostatic mechanisms that link vascular O_2_ supply to metabolic demand. Hypoxia plays a critical role in controlling both physiological and pathological conditions and advances in molecular characterization of angiogenic pathways identified HIF as a key transcriptional regulator able to induce new blood vessels. However, as shown in this minireview, the control of angiogenesis involves a more complex net of factors including, but not limited to, miRNAs, other cell types, exosomes, and metabolic adaptative responses to hypoxia. All of these may exploit common signaling pathways, that are often related to hypoxia. Thus, targeting these signaling pathways by novel approaches may be important in the development of therapeutic strategies in the pathological conditions where angiogenesis is compromised (Figure 2).
